# Driving following defibrillator implantation: development and pilot results from a nationwide questionnaire

**DOI:** 10.1186/s12872-018-0949-3

**Published:** 2018-11-20

**Authors:** Jenny Bjerre, Simone Hofman Rosenkranz, Anne Mielke Christensen, Morten Schou, Christian Jøns, Gunnar Gislason, Anne-Christine Ruwald

**Affiliations:** 1 Department of Cardiology, Cardiovascular Research, Copenhagen University Hospital Herlev-Gentofte, Kildegaardsvej 28, 2900 Hellerup, Denmark; 2 Research and Test Center for Health Technologies, Copenhagen University Hospital, Rigshospitalet-Glostrup, Valdemar Hansens Vej 1-23, 2600 Glostrup, Denmark; 3 Department of Cardiology, Copenhagen University Hospital Rigshospitalet-Glostrup, Blegdamsvej 9, 2100 Copenhagen Ø, Denmark; 4 Department of Medicine, Zealand University Hospital, Sygehusvej 10, 4000 Roskilde, Denmark

**Keywords:** Implantable cardioverter defibrillators, Driving restrictions, Traffic safety, Patient-reported outcomes, Questionnaire

## Abstract

**Background:**

Implantable cardioverter defibrillator (ICD) implantation is associated with driving restrictions which may have profound effects on the patient’s life. However, there is limited patient-reported data on the information given about driving restrictions, the adherence to the restrictions, the incidence of arrhythmic symptoms while driving, and the driving restrictions’ effect on ICD patients’ daily life and quality of life factors. A specific questionnaire was designed to investigate these objectives, intended for use in a nationwide ICD cohort.

**Methods:**

The conceptual framework based on literature review and expert opinion was refined in qualitative semi-structured focus group interviews with ten ICD patients. Content validity was pursued through pre-testing, including expert review and 28 cognitive interviews with patients at all ICD implanting centres in Denmark. Finally, the Danish Pacemaker and ICD registry was used to randomly select 50 ICD patients with a first-time implantation between January 1, 2013 and November 30, 2016 for pilot testing, followed by a test-retest on 25 respondents. Test-retest agreement was assessed using kappa statistics or intraclass correlation coefficients.

**Results:**

The pilot test achieved a response rate of 78%, whereof the majority were web-based (69%). Only 49% stated they had been informed about any driving restrictions after ICD implantation, whereas the number was 75% after appropriate ICD shock. Among respondents, 95% had resumed private driving, ranging from 1 to 90 days after ICD implantation. In those informed of a significant (≥ 1 month) driving ban, 55% stated the driving restrictions had impeded with daily life, especially due to limitations in maintaining employment or getting to/from work and 25% admitted they had knowingly been driving during the restricted period. There were six episodes of dizziness or palpitations not necessitating stopping the vehicle. Test-retest demonstrated good agreement of questionnaire items, with 69% of Kappa coefficients above 0.60.

**Conclusions:**

We have developed a comprehensive questionnaire on ICD patients’ perspective on driving. Pre-testing and pilot testing demonstrated good content validity, feasible data collection methods, and a robust response rate. Thus, we believe the final questionnaire, distributed to almost 4000 ICD patients, will capture essential evidence to help inform driving guidelines in this population.

**Electronic supplementary material:**

The online version of this article (10.1186/s12872-018-0949-3) contains supplementary material, which is available to authorized users.

## Background

Implantable cardioverter defibrillators (ICDs) are effective in preventing sudden cardiac death, both in patients who have survived a life-threatening arrhythmia (secondary prevention) and in patients who are at increased risk of life-threatening arrhythmias (primary prevention) [1–3]. However, since the early days of ICD treatment, driving following ICD implantation and ICD therapy has been controversial. The concern is that the underlying heart condition may cause an arrhythmia, potentially incapacitating the patient while operating a motor vehicle and causing harm to the patient or others. Hence, scientific societies have developed statements on the issue [4, 5]: The current European recommendations advise a four-week driving restriction following primary prevention ICD implantation and three-months restriction following secondary prevention ICD implantation as well as after appropriate ICD therapy [5]. In Denmark, primary prevention patients can resume driving 1 week following implantation, granted home-monitoring is established [6]. Professional driving and driving of large vehicles (> 3.5 metric tonnes) is permanently restricted [5, 6].

There is an overall paucity of research involving patient-reported outcomes in this area. The few available studies date back to the 1990s and were performed in small and selected patient populations, predominantly including secondary prevention patients. Notably, authors have not reported their methods for developing and testing the questionnaires and rarely presented the questionnaires themselves. These studies reported that information given on driving restrictions is often either lacking or cannot be recalled by the patients [7, 8], and even when instructed not to drive, adherence to the instructions is minimal [7–11]. Moreover, evidence suggest that driving restrictions following ICD implantation negatively impacts the patients’ quality of life (QoL) [12].

In questionnaire research, it is pivotal that the questions asked are both relevant and comprehensive for the objective of the study - a measure of content validity. Likewise, the questionnaire items should be understandable and unambiguous for the target population, warranting thorough pre-testing of the measure before final application. Lastly, data collection procedures should be evaluated through a pilot test, to secure sufficient and analyzable responses.

We aimed to develop a questionnaire investigating ICD patients’ experiences with driving and driving restrictions. The intended use was for a cross-sectional investigation in a nationwide Danish ICD cohort. This paper describes the process of developing and pre-testing the questionnaire and ultimately presents selected results from a pilot test.

## Methods

**Fig. 1:**
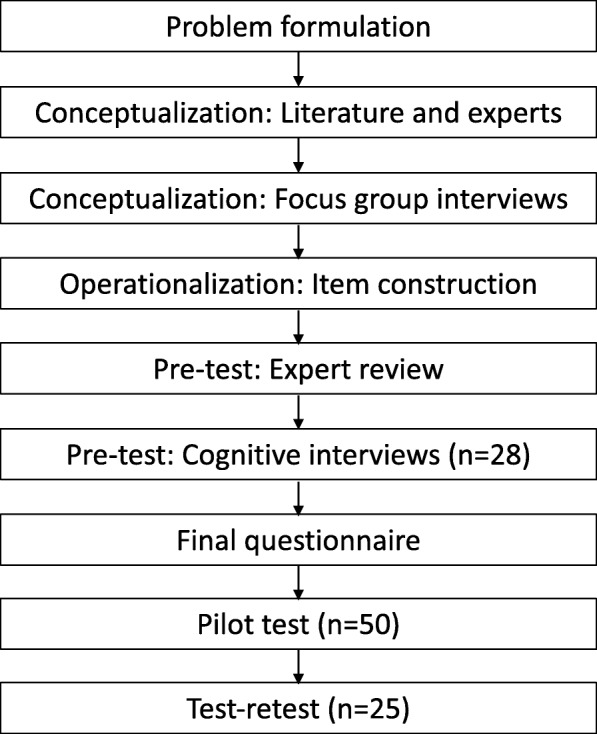
Overview of the questionnaire developmental process

The International Society for Quality of Life Research (ISO-QOL) recommendations for patient-reported outcome measures were used as a manual to guide questionnaire development and testing [13]. These recommendations have been developed with the aim to define minimum measurement standards to promote appropriate use of patient-reported outcomes research. Figure 1 demonstrates a flow chart of questionnaire development.

### Problem formulation

Initially, we defined four specific objectives for the questionnaire: (1) Quantify the amount of information given to ICD patients on driving restrictions following ICD implantation and/or ICD shock; (2) Investigate whether ICD patients adhere to driving restrictions and which factors are associated with adherence to driving restrictions; (3) Determine what proportion of Danish ICD patients have experienced an ICD shock or cardiac symptoms of possible arrhythmia while driving, and whether these symptoms resulted in a motor vehicle accident; and (4) Identify whether driving restrictions influence factors associated with patient QoL.

### Conceptualization: Literature and experts

Based on expert opinion and following review of the existing literature on driving following ICD implantation, an initial conceptual framework was developed which was successively refined in qualitative semi-structured focus group interviews. In summary, through conceptualization, specific variables were defined which could subsequently be transformed into items, thus connecting the research objectives with the content of the questionnaire. Additional file 1: Table S1 presents our research aims, hypotheses, concepts and their corresponding variable definitions as well as questionnaire item numbers. For example, the concept “Information about driving restrictions after ICD implantation” resulted in three variable definitions: (1) Whether the patient held a valid private driver’s license (Group 1: car, motorcycle, tractor) during the 6 months leading up to ICD implantation; (2) Whether the patient held a valid Group 2 driver’s license (Group 2: truck, bus or any vehicle for passenger transportation) during the 6 months leading up to ICD implantation; (3) Whether the patient was informed about driving restrictions following ICD implantation (for both Group 1 and Group 2) by health personnel (doctors, nurses and ICD technicians) during the hospitalization for ICD implantation.

A few concepts and variable definitions merit further elaboration: First, in order to compare questionnaire responses with current guideline recommendations, we chose to define private and professional driving based on the definitions in the European Heart Rhythm Association’s recommendations for driving with ICDs and adapted by the Danish Society of Cardiology [5, 6]. For our aim of investigating the driving restrictions’ influence on QoL factors, we were inspired by the World Health Organization’s six proposed domains of QoL [14]. From the 24 facets of QoL proposed, we predicted that 10 could be affected by driving restrictions: negative feelings, positive feelings and self-esteem (psychological domain); mobility, activities of daily living and work capacity (level of independence domain); personal relationships and social support (social relationship domain); participation in and opportunities for recreation/leisure and transport (environment domain).

### Conceptualization: Focus group interviews

To uncover unknown concepts related to driving following ICD implantation, we performed three focus group interviews with ten contemporary Danish ICD patients (20% female, median age 62 years, 50% primary prevention ICD indication). The participants were recruited from the outpatient clinic at a university hospital in The Capital Region of Denmark and purposive maximum sampling was used to achieve variation in pre-specified variables, including sex, age, ICD indication, previous ICD therapy and geographic residence. Briefly, the qualitative setting allowed ICD patients to elaborate on their perceptions and experiences with driving and the driving restrictions. Further, we could observe the social and cultural norms within groups of ICD patients and thus gain essential knowledge on what wording to use and how to approach certain delicate questions, such as adherence to driving restrictions [15].

### Operationalization

We followed general technical rules for valid questionnaire design. Initially, the items were grouped into themes using a strategic order, taking into account spill over effects and placement of sensitive questions. Thus, the first questions were considered easy, whereas complexity increased throughout the questionnaire. Respondents were asked when they had resumed driving after ICD implantation or ICD shock, before the questions about what information on driving restrictions they had received. Intentionally, direct questions about adherence to driving restrictions were placed at the end of the questionnaire. Since not all questions were relevant for everyone, e.g. the majority of ICD patients have not experienced an ICD shock, branching methods were applied to guide the respondents through the questionnaire. For instance, following a few background questions (about self-assessed health, educational attainment, and employment status), respondents without a valid driver’s license at time of ICD implantation were guided to the end of the questionnaire. Lastly, due to the sensitive nature of some of the questions, we chose to make all items voluntary including a possibility of skipping questions in the web-based questionnaire.

Question formulations were kept short and words with connotations were excluded. Help texts were included if deemed necessary*.* In order to minimize recall bias, we defined four distinct recall periods (which were confirmed realistic during focus groups) depending on the objective of the question: “the six months leading up to ICD implantation”, “at the time of ICD implantation”, “in periods with driving restrictions”, and “during the previous month.” Text including recall periods was underscored. Response categories were tailored to the question type: for factual questions, we particularly aimed for exhaustive and mutually exclusive response categories, whereas the focus for opinion questions was to achieve balance in the response categories. For potentially sensitive questions, including questions about driving behaviour and the driving restrictions’ influence on factors related to QoL, we chose to express questions as opinions with corresponding Likert scale response categories (strongly agree to strongly disagree) [16], as opposed to factual questions with Yes/No response categories. Likert scale variation was kept minimal with either three- or five-point scales. Likewise, open response categories were generally avoided, but included in a few “other” response categories. Furthermore, the respondents were given the opportunity to leave comments after selected sections of the questionnaire.

### Pre-test: Expert review

The first method used in evaluating the questionnaire was an informal, individually-based expert review. Four participating cardiologists or cardiology fellows (ACR, MS, JP and LS) and one device technician (JDP) each independently conducted a review and determined whether he/she found the items sufficient relative to the questionnaire’s aims, or if a questionnaire item was problematic. Following adjustments based on these evaluations, the questionnaire was reviewed by a senior questionnaire expert (JC), independent from the study group. Adjustments following this expert review included minor modifications like introducing help texts to emphasize different recall periods, using continuous response categories for questions on time to resumption of driving following ICD implantation and/or shock, and strongly urging respondents to complete the questionnaire with a next of kin.

### Pre-test: Cognitive interviews

Further examination of content validity and refinement of items was done by cognitive interviewing of 28 ICD patients in the outpatient clinic at all six ICD implanting centres in Denmark (range: 4–6 patients/centre). We chose to include ICD patients from all five regions of Denmark to capture any geographic variance potentially influencing questionnaire responses (Capital Region: 8 participants; Zealand Region: 4 participants; Southern Denmark: 5 participants; Central Jutland: 6 participants; Northern Jutland: 5 participants). On a given day, ICD patients visiting the outpatient clinic were approached in a random manner and were excluded only if they did not have a valid driver’s licence at time of ICD implantation. A retrospective verbal probing approach was used, and the probe questions were asked following completion of each of the four sections of the questionnaire. Probes were both scripted (e.g. “What does the term ICD shock mean to you?”) and spontaneous (e.g. “I noticed that you hesitated. Can you tell me what you were thinking?”). We utilized Tourangeau’s 4-stage cognitive model, investigating: (1) comprehension of the question; (2) retrieval of information (recall strategy); (3) decision processes; and (4) response processes [17]. Another significant focus in these cognitive interviews was recognizing any reluctance in answering questions about adherence to driving restrictions and further, to identify what wording to use to make respondents trust that information on non-adherence would remain anonymous.

Head-investigator (JB) performed all the cognitive interviews over a period of 16 weeks as an iterative process with alterations of the questionnaire items where necessary after each interview session. The initial five rounds of cognitive interviews were conducted using the web-based questionnaire, while the patients in the last round of interviews were presented with the paper format questionnaire. After the cognitive interviews at each implanting centre were completed, JB reviewed and summarized the results and determined whether, for each tested item, significant problems had been detected. Throughout the interviewing process, the frequency of problems encountered per patient interviewed, declined (Additional file 2: Table S2).

### Pilot test

The nationwide Danish Pacemaker and ICD registry was used to identify all individuals with a first-time ICD implantation in the period January 1, 2013 to November 30, 2016. Among these, 50 individuals were randomly selected to participate in the pilot test and were subsequently matched with up-to-date address data from the Danish Civil Person Register. Invitations to participate in the study were mailed on February 23, 2017. Participants were urged to complete the web-based questionnaire, however, an option to request a paper version was presented (Additional file 3, translated from Danish). Following 3 weeks, a reminder was mailed to all non-responders including a paper version of the questionnaire and a prepaid return envelope. The web-based questionnaire software SurveyXact, developed by Rambøll A/S and approved for research purposes by the Capital Region of Denmark and the Danish Data Protection Agency, was used for data collection [18]. The pilot test was terminated on April 13, 2017, after 7 weeks’ data collection. All paper responses were entered into the web-based questionnaire software by double manual data entry to reduce the consequences of potential human errors.

### Test-retest

To investigate the reproducibility of the questionnaire items, we invited the first 25 pilot test participants who responded to the questionnaire to complete the questionnaire again at 3 weeks after their first response. This timeframe was deemed appropriate as to avoid memory effects positively influencing the test-retest reliability. No reminders were distributed to test-retest participants.

### Statistical analyses

Results of the pilot test were summarized by reporting responses on selected questionnaire items by available case analysis, thus, the number of responses to each question may vary. For descriptive analyses, categorical variables are reported as percentages and continuous variables are presented as medians with interquartile range. Test-retest agreement was investigated by kappa coefficients or weighted kappa coefficients for categorical variables and intraclass correlation coefficients for continuous variables. All analyses were performed using SAS (version 9.4, Cary, NC, USA).

## Results

### Focus group interviews

All concepts already identified from the literature were confirmed in the focus groups interviews, excluding the effect of driving restrictions on self-esteem. In addition, we discovered that many of the participants had changed their driving behaviour in some way after ICD implantation: For example, some patients reported being extra cautious when driving with children (e.g. driving at slower speed, avoiding the overtaking lane on highways) or avoiding highways altogether. Also, a significant proportion of primary prevention patients called for more information about potential driving restrictions in case of future ICD therapy and also being explained the rationale behind the driving restrictions, as they saw these as promoting factors for compliance with the restrictions. Generally, in an anonymous setting, the participants willingly volunteered information about not adhering to the driving restrictions.

### Pilot test

**Fig. 2:**
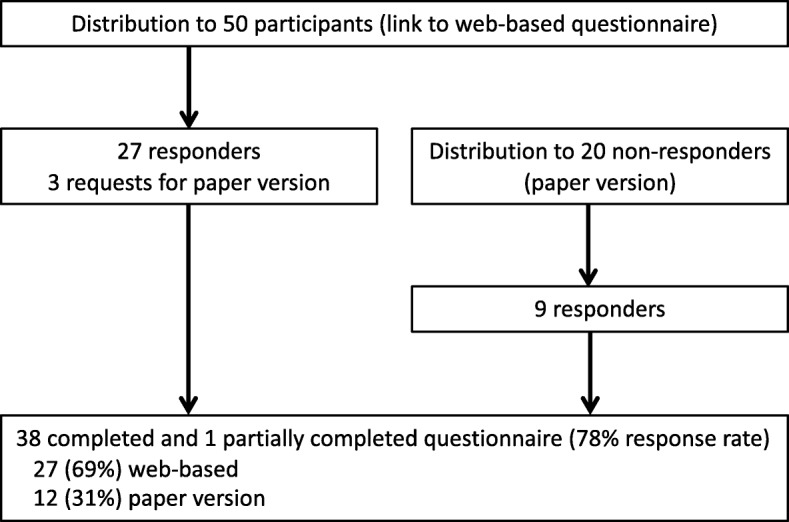
Pilot test flow chart. Overview of distribution of the questionnaire. Initial distribution included a patient-specific link to the web-based questionnaire, whereas the second distribution additionally included a paper version of the questionnaire

Among the 50 ICD patients (9 women (18%)) invited to participate in the pilot test, 30 (60%) either responded electronically or requested a paper version of the questionnaire within 3 weeks. Following only one reminder, 39 participants (78%) completed the questionnaire (Fig. 2). Almost all web-based responses (85%) came within 1 week. Median time for completing the questionnaire was 9.5 min for the web-based responses (IQR: 5.2–13.7 min).

**Fig. 3:**
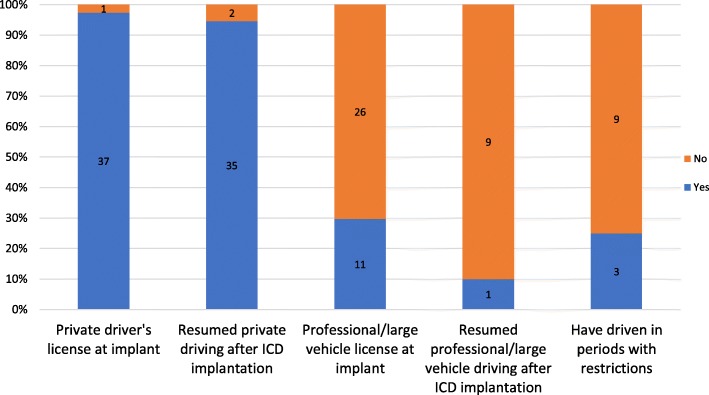
Driving characteristics before and after ICD implantation. Overview of respondents with private and professional/large vehicle driver’s licences before ICD implantation, their resumption of driving following ICD implantation as well as non-adherence to the driving restrictions (defined as restrictions of minimum 1 month’s duration following either ICD implantation or ICD therapy)

Of the responders, 37 (97%) held a valid private driver’s license for car, motorcycle or tractor (Group 1 drivers) prior to implantation and 11 (30%) held a valid driver’s license for large vehicles or any professional driving (Group 2 drivers) (Fig. 3). However, only two responders had actively used their professional license during the 6 months prior to ICD implantation – both were truck drivers. The most common occasions (> 75%) for driving prior to ICD implantation included practical errands, visits to family and friends and in relation with leisure activities, while eight (22%) drove during work hours (Table 1).

**Table 1:** Driving characteristics before ICD implantation

Driving characteristics	No. of patients (%)
Private driver’s license (*n* = 38)	37 (97%)
Professional/large vehicle driver’s license (*n* = 37)	11 (30%)
Purpose of professional/large vehicle driver’s licence (*n* = 10)	
Truck driver	4 (40%)
Bus driver	2 (20%)
Taxi driver	0
Other, including private use	4 (40%)
Use of professional/large vehicle driver’s licence six months prior to implantation (*n* = 10)	2 (20%)
Other members of the household hold driver’s license (*n* = 37)	26 (70%)
Typical occasions for driving six months prior to implantation (*n* = 37)	
Professional driving or driving of large vehicle during work hours	2 (5%)
During work hours	8 (22%)
To/from work/school	16 (43%)
In relation with practical errands	31 (84%)
To visit family and friends	33 (89%)
In relation with leisure activities	28 (76%)
Did not drive during the period	2 (5%)
Average hours/week driving prior to implantation (*n* = 35)	
< 1 h a week	0
1–3 h a week	12 (34%)
4–6 h a week	7 (20%)
7–9 h a week	7 (20%)
10 h a week or more	9 (26%)

Only half of the respondents remembered being informed of any driving restrictions for private driving. Of these, two thirds reported they had solely been informed verbally (Table 2). The information given by health personnel on driving following implantation as recalled by the patients ranged from resuming to drive immediately after ICD implantation to never to drive again. Still, patients were overall satisfied with the communication of the driving restrictions. Among the responders actively driving professionally prior to ICD implantation, only one (50%) could remember being told never to drive professionally again. Of the ICD patients having experienced an appropriate ICD shock, three out of four remembered having been verbally informed not to drive for 3 months.

**Table 2:** Information on driving restrictions following ICD implantation, as reported by patients

Variable	No. of patients (%)
Informed about driving restrictions (*n* = 37)	
Yes	18 (49%)
No	7 (19%)
Cannot remember	12 (32%)
Way information was given (*n* = 18)	
Only verbally	12 (67%)
Only in writing	–
Both verbally and in writing	6 (33%)
Information given (*n* = 18)	
Resume driving immediately	1(6%)
Resume when I felt ready	1 (6%)
Resume after 1 week	4 (22%)
Resume after 1 month	3 (17%)
Resume after 3 months	8 (44%)
Resume after 6 months	–
Never to drive again	1 (6%)
Level of satisfaction with information given (*n* = 18)	
Very satisfied or satisfied	14 (78%)
Neutral	4 (22%)
Dissatisfied or very dissatisfied	–

Following ICD implantation, 35 (95%) of responders with a driver’s license resumed private driving whereas professional driving was resumed by one responder (Fig. 3). Overall median time to resumption of driving was 14 days (interquartile range: 2–60) while professional driving was resumed at 8 months. Among patients being instructed of a minimum 1-month driving ban, either after ICD implantation or ICD shock, three patients (25%) admitted they had been driving although restricted. None had experienced an ICD shock or loss of consciousness while driving, but two patients (6%) had experienced dizziness and four patients (11%) had experienced palpitations while driving. None of these events necessitated stopping the vehicle or resulted in a motor vehicle accident.

Overall, patients had not altered their driving behaviour after ICD implantation. Only four patients (12%) were afraid of having an ICD shock while driving, and only one patient was nervous about driving or tried to avoid highways as a consequence of ICD implantation. None avoided driving alone or driving with children in the car. Among the 11 patients with a significant driving restriction (defined as ≥1 month), six (55%) stated that the driving restrictions in some way had impeded with their daily life and 45 and 33% responded that they had been very limited in maintaining employment and getting to/from work, respectively.

### Test-retest

Of the 25 responders invited to participate in a test-retest questionnaire, 19 (75%) responded within 3 weeks. Kappa statistics found that 69% of the analyzed questionnaire items had substantial agreement, with kappas or weighted kappas above 0.6, whereof 37.5% were above 0.8. (Additional file 4: Table S3). Generally, the kappas with moderate (0.4–0.6) and fair agreement (0.2–0.4) were found in the section on the driving restrictions’ influence on factors associated with QoL. In one item, the only test-retest response was reverse, rendering a kappa of 0. The intraclass correlation coefficient for time to resumption of private driving following ICD implantation and appropriate ICD shock was 0.98 and 1.00, respectively, whereas the time to resumption of professional driving could not be analyzed as the item only had one test-retest response.

## Discussion

Through a thorough developmental and testing process, we have constructed a questionnaire aimed to investigate ICD patients’ perspective on driving, including the information received on driving following ICD implantation and/or ICD shock, adherence to the driving restrictions, episodes of cardiac symptoms while driving, the driving restrictions’ influence on factors associated with QoL, and alterations of driving behaviour after ICD implantation. The pilot test administered to 50 randomly selected Danish ICD patients achieved a response rate of 78% over seven-week period with only one reminder. The high response rate confirms our understanding that this is an area of great importance to ICD patients and furthermore implies a high level of target population comprehension of the developed questionnaire.

Our pilot test results support previous research reporting poor communication between health professionals and ICD patients on the subject of driving [7, 9, 19]: 20% reported they had not received any information on driving restrictions after ICD implantation. However, in the final questionnaire it will be important to stratify these data on both ICD indication, as well as time since ICD implantation to account for time as a recall bias. In fact, 30% of responders reported they could not remember if they had been informed on any driving restrictions. Nevertheless, given that professional guidelines recommend driving restrictions after ICD implantation and ICD therapy, it is pivotal for patient compliance that these restrictions are communicated to the patients. A recent study found that a systematic counselling program before patient discharge had a positive impact on both patient compliance with treatment, as well as patient QoL [20]. Similar effects could be anticipated in terms of compliance with driving restrictions and our results will quantify the need for potential improvements in this area.

In the focus group interviews, some primary prevention ICD patients called for information at the time of ICD implantation on potential driving restrictions in case of future ICD shocks. However, to reduce respondent burden and because we considered the concepts would be more suitable for investigation in a qualitative method, we chose not to include these concepts in the final questionnaire.

Previous studies suggest many ICD patients resume driving earlier than instructed [7, 8, 10, 11]. Prior to developing the questionnaire, we had concerns regarding the patients’ willingness to answer questions on sensitive matters such as non-adherence to driving restrictions. However, throughout the developmental process including both focus group and cognitive interviews, we found that this was not a substantial problem. Following recommendations from pre-test participants, we included multiple statements affirming that information would not be forwarded to the authorities. In the pilot test, 25% of responders with a significant driving ban admitted they had been driving while restricted. This is comparable to previously published results [8], thus, we feel assured that respondents not answering truthfully is not a major issue. To capture both ICD patients knowingly and unknowingly driving during the guideline-recommended restricted period, we also included questions about time to resumption of both private and professional driving after ICD implantation, as well as after ICD shock. For example, one Group 2 driver who believed he had not received information on restrictions for professional driving, stated that this had been resumed 8 months following implantation.

This questionnaire was not designed to measure overall QoL in an ICD population, but rather to investigate if the driving restrictions themselves had influenced factors associated with QoL. Particularly, the risk of losing the ability to earn an income as a professional driver has not been systematically investigated previously. Thus, we included a separate question to professional drivers on whether they had lost their job as a result of the driving restrictions. In the small pilot test sample, this was not the case for any of the two truck drivers. However, more than half of the respondents with a driving restriction longer than 1 month said the restrictions had impeded with their daily life, specifically maintaining employment and getting to/from work. Thus, the driving restrictions can definitely temporarily restrict ICD patients’ ability to work and thus affect their household economic status, even if they are not professional drivers.

Focus groups revealed that some ICD patients alter their driving behaviour because of ICD implantation in other aspects than quantity of time spent driving. Consequently, we chose to include questions on this specific aim in the questionnaire. However, in the small pilot sample, only 12% expressed concerns about having an ICD shock while driving and only one respondent expressed avoidance behaviour with regards to driving on highways. By linking the final questionnaire with the ICD registry, the future results of the questionnaire can relevantly be stratified by ICD indication as well as history of ICD shocks.

The pilot test achieved a response rate of 78%, and impressively, more than two-thirds of the responses were web-based. This number was higher than anticipated in a population with a mean age around 62 years [21], and decreased the investigator’s risk of making typing mistakes as well as the burden of manually entering paper responses into the web-based questionnaire database. Further, web-based questionnaires in general reduce respondent burden as the completed questionnaires do not need to be mailed. To capture a wide range of the target population and not exclude individuals with limited computer skills, we chose to move forward with both modalities of data collection.

Test-retest found almost perfect agreement in nearly 40% of the items and substantial agreement in 31% of all items [22]. The generally high reliability indicates that, despite the high mean age and disease burden in the population, the responses produced by the questionnaire are representative and stable over time. However, the limit for when kappa statistics are in sufficient agreement are both subjective and arbitrary, and furthermore, our small sample of 19 test-retest respondents should be considered. Besides, due to the branching of the questionnaire, we were unable to retest all items regarding rare events, for example the items on cardiac symptoms while driving.

### Study limitations and future perspectives

Questionnaire studies pose some significant inherent limitations. Many relate to poor question design (e.g. problems with wording, leading questions, scale formats) or questionnaire design (e.g. formatting problems, too long or complex questionnaire) which we have tried to overcome by thorough pre-testing, but some questions will inevitably be misinterpreted. Moreover, though the focus group participants were generally good at recalling specific information from the time of ICD implantation, recall bias cannot be excluded. Also, social desirability may contribute to respondents giving untrue answers, particularly to the questions related to adherence, although focus group and cognitive interviews demonstrated the contrary. Lastly, we naturally only included patients who were alive at the time of questionnaire administration, introducing a healthy participant bias which, similarly to the non-response bias, covers the fact that responders (or survivors) could differ significantly from the non-responders.

The questionnaire was developed specifically for use in a nationwide cohort of Danish ICD patients and the final questionnaire has been administered to more than 3900 individuals. Due to the unique Danish administrative registries, we will be able to link the final questionnaire results with reliable clinical and demographic information, including indication for ICD implantation, socio-economic status, and comorbidities. Importantly, linkage with the nationwide registries also allows comparison between responders and non-responders, a major strength in a questionnaire study.

In this pilot study, however, the results have not been linked with relevant clinical data and we were therefore not able to stratify results on important clinical information such as cardiac resynchronization status, pharmacological treatment and comorbidities such as diabetes. All of these will be relevant to include in the final questionnaire study when investigating factors associated with the risk of ICD therapy while driving, as they have been found to affect the risk of adverse clinical outcomes, including ICD therapy [21, 23–25]. Other limitations of the current study include lack of information on heart failure biomarker levels and home monitoring status which are also expected to affect clinical outcomes, especially in primary prevention patients with a cardiac resynchronization device [26–28].

## Conclusion

In summary, we have developed a comprehensive questionnaire on the patient’s perspective on driving and driving restrictions following ICD implantation, with good content validity and acceptable patient and investigator burden. From pilot test results, we anticipate that the final questionnaire, distributed to > 3900 ICD patients, will very likely reach a response rate above 60% and provide much-needed data about information on, adherence to and impact of driving restrictions in a large, nationwide, contemporary cohort of ICD patients. We believe these results will be valuable and potentially identify problematic areas in need of further focus or interventions.

## Additional files

Additional file 1: **Table S1.** Questionnaire Conceptualization Process: The research aims, hypotheses, concepts and corresponding variable definitions [7–12, 19, 29–33]. (DOCX 90 kb)

Additional file 2: **Table S2.** Frequency of Problems Identified During Cognitive Interview Sessions. (DOCX 16 kb)

Additional file 3: “Danish ICD Patients’ Perspective on Driving – a Nationwide Survey. 2017”. English version (translated from Danish) of the questionnaire specifically constructed for this research project. (PDF 927 kb)

Additional file 4: **Table S3.** Test-Retest Agreement. (DOCX 19 kb)

## Abbreviations

ICD: Implantable Cardioverter Defibrillator
QoL: Quality of Life
