# Supplementary material for: Driving following defibrillator implantation: development and pilot results from a nationwide questionnaire
Source: BMC Cardiovasc Disord. 2018 Nov 20;18:212. doi: 10.1186/s12872-018-0949-3 (PMC6245910; doi:10.1186/s12872-018-0949-3)
Supplement: Supplementary file 1 — Table S1. Questionnaire Conceptualization Process: The research aims, hypotheses, concepts and corresponding variable definitions [7–12, 19, 29–33]. (DOCX 90 kb) [file 12872_2018_949_MOESM1_ESM.docx]

**Additional file 1: Table S1 Questionnaire Conceptualization Process: The research aims, hypotheses, concepts and corresponding variable definitions**

| **Aim 1: Quantify the amount of information given to ICD patients on driving following ICD implantation and/or ICD shock** | | | |
| --- | --- | --- | --- |
| **Concept 1: Information about driving restrictions after ICD implantation** | **Sources** | **Variable definition** | **Item no.** |
| *Not all ICD patients are given information about driving restrictions following ICD implantation* | Literature  [7-9, 19]  Expert opinion  Focus groups | Whether the patient held a valid private driver’s license (Group 1: car, motorcycle, tractor) during the six months leading up to ICD implantation | A4 |
|  |  | Whether the patient held a valid Group 2 driver’s license (Group 2: truck, bus or any vehicle for passenger transportation) during the six months leading up to ICD implantation | A5 |
|  |  | Whether the patient was informed about driving restrictions following ICD implantation (for both Group 1 and Group 2 driving) by health personnel (doctors, nurses and ICD technicians) during the hospitalization for ICD implantation | A18, C4 |
| *The information is not always given both in person and in writing* | Expert opinion  Focus groups | The mode of communicating the information about the driving restrictions following ICD implantation (in writing, in person, or both) | A18.1, C4.1 |
| *The information given does not always comply with the guidelines* | Literature  [7, 9, 19]  Focus groups | The specific information given about driving restrictions following ICD implantation, as remembered by the patient | A18.2, C4.2 |
| *The ICD patient does not always receive information about driving restrictions in case of future ICD therapy* | Focus groups | Whether the patient, at time of ICD implantation, was given any information about potential driving restrictions in case of future ICD shock | Not included |
| *ICD patients who receive information about driving restrictions find them insufficient* | Focus groups | The ICD patient’s level of satisfaction with the communication of the driving restrictions | A19, C6 |
| **Concept 2: Information about driving restrictions after ICD shock** |  | | |
| *Not all ICD patients who experience an ICD shock are given information about driving restrictions following ICD shock* | Expert opinion  Focus groups | Whether the patient has experienced minimum one ICD shock | A7 |
|  |  | Whether the experienced shock/shocks was/were appropriate (to terminate a life-threatening arrhythmia), inappropriate, or both | B1 |
|  |  | Whether the patient’s most recent appropriate shock occurred more or less than 3 months ago | B2 |
|  |  | Whether the patient communicated (by telephone or in person) with the ICD clinic following the appropriate ICD shock? | Not included |
|  |  | Whether the patient was informed by health personnel (doctors, nurses and ICD technicians) about driving restrictions following appropriate ICD shock | B4 |
| *The information is not always given both in person and in writing* | Expert opinion  Focus groups | The mode of communicating the information about the driving restrictions following appropriate ICD shock (in writing, in person, or both). | B4.1 |
| *The information given does not always comply with the guidelines* | Literature [7]  Focus groups | The specific information given about driving restrictions following appropriate ICD shock, as remembered by the patient | B4.2 |
| *Patients who receive information about driving restrictions find them insufficient* | Focus groups | The patient’s level of satisfaction with the communication of the driving restrictions following appropriate ICD shock | B5 |

| **Aim 2: Investigate whether ICD patients adhere to the driving restrictions and which factors are associated with adherence** | | | | |
| --- | --- | --- | --- | --- |
| **Concept 1: Do ICD patients adhere to the driving restrictions?** | **Sources** | | **Variable definition** | **Item no.** |
| *Some ICD patients resume private driving while still restricted following ICD implantation* | Literature  [7, 10, 11]  Expert opinion  Focus groups | | Whether the patient has resumed private (Group 1) driving following ICD implantation | A10 |
|  |  |  | How long after ICD implantation the patient resumed private driving | A10.1 |
| *Some ICD patients resume professional driving after ICD implantation* | Expert opinion | | Whether the patient has resumed professional (Group 2) driving following ICD implantation | C3 |
|  |  |  | How long after ICD implantation the patient resumed professional driving | C3.1 |
| *Some ICD patients resume private driving while still restricted following appropriate ICD shock* | Expert opinion  Focus groups | | Whether the patient has resumed private (Group 1) driving following his/her first appropriate ICD shock | B3 |
|  |  |  | How long after the patients first appropriate ICD shock the patient resumed private driving | B3.1 |
| *Some ICD patients find it hard to adhere to driving restrictions of minimum 1 months duration* | Literature [12]  Focus groups | | Whether the patient have found it difficult to adhere to the driving restrictions | A23 |
| *Some ICD patients drive knowingly while restricted* | Literature [8, 10]  Expert opinion  Focus groups | | Whether the patient has been driving in periods (of minimum 1 months duration) in which he/she had been informed that he/she was restricted from driving | A24 |
| **Concept 2: Factors associated with adherence to the driving restrictions** | |  | |  |
| *ICD patients who drove frequently before ICD implantation are less likely to have adhered to the driving restrictions* | Literature  [9, 10, 12, 29, 30]  Focus groups | | The patient’s average driving habits six months prior to ICD implantation, both occasions for driving (professional, during work hours, to work/school, for practical errands, social activities, leisure activities) and hours/week | A8, A9 |
|  |  |  | The patients current driving habits (hours/week) | A11 |
| *ICD patients living alone or without any other drivers in the household are less likely to have adhered to the driving restrictions* | Literature [10, 11]  Focus groups | | Whether anyone else in the patient’s household held a valid driver’s license at time of ICD implantation | A6 |
| *ICD patients with higher educational attainment are less likely to have adhered to the driving restrictions* | Expert opinion | | The patient’s highest educational attainment at time of questionnaire completion | A2 |
| *ICD patients who were employed at time of ICD implantation are less likely to have adhered to the driving restrictions* | Literature [10]  Focus group  Expert opinion | | The patient’s employment status at time of ICD implantation, disregarding any short sickness leave in conjuction with ICD implantation | A3 |
| *The patient’s conception about the legal consequences of driving while restricted influence their adherence to the driving restrictions* | Focus groups | | The patient’s evaluation of whether the fact that the police are not informed of any medical driving restrictions affected his/her adherence to the driving restrictions | Not included |
| *ICD patients with better self-assed health are less likely to adhere to the driving restrictions* | Expert opinion  Focus groups | | The patient’s description of his/her general health  (From the 36-item Short Form Health Survey, SF-36). | A1 |

| **Aim 3: Determine what proportion of Danish ICD patients have experienced an ICD shock or cardiac symptoms of possible arrhythmia while driving, and whether these symptoms resulted in a motor vehicle accident** | | | | |
| --- | --- | --- | --- | --- |
| **Concept 1: Experienced ICD shock while driving** | **Sources** | | **Variable definition** | **Item no.** |
| *Very few ICD patients have experienced an ICD shock while driving a motor vehicle and further, very few of these ICD shocks resulted in loss of consciousness or a motor vehicle accident* | Literature  [9-11, 31, 32]  Expert opinion | | Whether the patient has experienced an ICD shock while driving a motor vehicle (car, motorcycle, truck, tractor or bus) | A12 |
|  |  |  | How many times the patient has experienced an ICD shock while driving a motor vehicle | A12 |
|  |  |  | Whether the patient lost consciousness in conjunction with the ICD shock received while driving | A13 |
|  |  |  | Whether the ICD shock resulted in a motor vehicle accident (leading to physical injury or material damage) | A13.1 |
| **Concept 2: Experienced symptoms of possible arrhythmia while driving** | |  | |  |
| *Very few ICD patients have lost consciousness while driving a motor vehicle and further, very few of these events resulted in a motor vehicle accident* | Literature  [8, 9, 33]  Expert opinion | | Whether the patient has experienced loss of consciousness while driving a motor vehicle (car, motorcycle, truck, tractor or bus) | A12 |
|  |  |  | How many times the patient has lost consciousness while driving a motor vehicle | A12 |
|  |  |  | Whether the loss of consciousness resulted in a motor vehicle accident (leading to physical injury or material damage) | A14 |
| *Very few ICD patients have experienced other cardiac symptoms while driving a motor vehicle and further, very few of these events resulted in a motor vehicle accident* | Literature [8, 9] Expert opinion | | Whether the patient has experienced dizziness, palpitations or chest pain while driving a motor vehicle (car, motorcycle, truck, tractor or bus) | A12 |
|  |  |  | How many times the patient has experienced dizziness, palpitations or chest pain while driving a motor vehicle | A12 |
|  |  |  | Whether the dizziness, palpitations or chest pain necessitated stopping the vehicle | A15, A16, A17 |
|  |  |  | Whether the dizziness, palpitations or chest pain resulted in a motor vehicle accident (leading to physical injury or material damage) | A15.1, A16.1, A17.1 |
| **Aim 4: Identify whether driving restrictions influence factors associated with patient quality of life (QoL)** | | | | |
| **Concept: Driving restrictions influence on factors associated with QoL** | **Sources** | | **Variable definition** | **Item** |
| *Overall, patients find that driving restrictions following ICD implantation and ICD shock impede with their lives* | Literature  [9, 10, 12, 29, 30]  Focus groups | | The patient’s evaluation of, in periods of minimum one month’s driving restriction, to which degree the driving restrictions have impeded with his/her life | A21 |
| *Driving restrictions following ICD implantation and ICD shock can influence the patients’ maintenance of social relations* | Literature  [10, 12, 29, 30]  Focus groups | | The patient’s evaluation of, in periods of minimum one month’s driving restriction, to which degree the driving restrictions have restricted his/her social relations with friends and family | A22 |
| *Driving restrictions following ICD implantation and ICD shock can influence the patients’ level of independence and activities of daily living* | Literature  [10, 12, 29, 30]  Focus groups | | The patient’s evaluation of, in periods of minimum one month’s driving restriction, to which degree the driving restrictions have restricted his/her ability to run errands | A22 |
|  |  |  | Whether the patient thinks the driving restrictions (of minimum 1 months duration) made him/her feel like a burden on his/her surroundings | A23 |
| *Driving restrictions following ICD implantation and ICD shock can influence the patients’ engagement in leisure activities* | Literature  [10, 12, 29, 30]  Focus groups | | The patient’s evaluation of, in periods of minimum one month’s driving restriction, to which degree the driving restrictions have restricted his/her ability to engage in leisure activities | A22 |
| *Driving restrictions following ICD implantation and ICD shock can influence the patients’ ability to work* | Literature [10, 29]  Focus groups  Expert opinion | | The patient’s evaluation of, in periods of minimum one month’s driving restriction, to which degree the driving restrictions have restricted his/her ability to maintain employment and getting to/from work/school | A22 |
| *Some ICD patients lose their job due to restrictions for professional driving following ICD implantation* | Expert opinion | | Whether the patient utilized his/her Group 2 license during the six months prior to ICD implantation | C2 |
|  |  |  | For which purpose the patient held a Group 2 licence during the six months prior to ICD implantation | C1 |
|  |  |  | Whether the patient lost his/her job due to professional (Group 2) driving restrictions following ICD implantation | C5 |
| *Driving restrictions following ICD implantation and ICD shock can influence the patients’ mood* | Literature [12, 29]  Focus groups | | Whether the patient thinks the driving restrictions (of minimum 1 months duration) affected his/her mood | A23 |
| **Aim 5: Identify whether ICD patients change driving behaviour following ICD implantation** | | | | |
| **Concept: Changed driving behaviour after ICD implantation** |  | | |  |
| *Some ICD patients are nervous about driving and afraid of receiving an ICD shock while driving* | Literature [7]  Focus groups | | Whether the ICD patient, as a result of having an ICD, is nervous about driving | A20 |
|  |  |  | Whether the ICD patient is afraid of having an ICD shock while driving |  |
| *Some ICD patients change their driving habits after ICD implantation* | Literature  [7, 9, 29, 32]  Focus groups | | Whether the ICD patient, as a result of having an ICD, tries to avoid highways | A20 |
|  |  |  | Whether the ICD patient, as a result of having an ICD, tries to avoid driving alone |  |
|  |  |  | Whether the ICD patient, as a result of having an ICD, tries to avoid driving with children |  |
|  |  |  | Whether the ICD patient, as a result of having an ICD, has altered his/her driving in any other way |  |
