# Supplementary material for: Driving following defibrillator implantation: development and pilot results from a nationwide questionnaire
Source: BMC Cardiovasc Disord. 2018 Nov 20;18:212. doi: 10.1186/s12872-018-0949-3 (PMC6245910; doi:10.1186/s12872-018-0949-3)
Supplement: Supplementary file 2 — Table S2. Frequency of Problems Identified During Cognitive Interview Sessions. (DOCX 16 kb) [file 12872_2018_949_MOESM2_ESM.docx]

Additional file 2: **Table S2** Frequency of Problems Identified During Cognitive Interview Sessions.

|  | **Interview session no. 1**  **(n=4)** | **Interview session no. 2**  **(n=5)** | **Interview session no. 3**  **(n=5)** | **Interview session no. 4**  **(n=6)** | **Interview session no. 5**  **(n=4)** | **Interview session no. 6**  **Paper version**  **(n=4)** |
| --- | --- | --- | --- | --- | --- | --- |
| **COMPREHENSION** | | | | | | |
| **Question content** | | | | | | |
| Vague topic/unclear question | 2 | 3 | 2 |  | 1 |  |
| Complex topic | 1 | 1 |  |  |  |  |
| Undefined/vague term | 2 |  | 4 | 1 | 4 |  |
| Potentially sensitive |  | 1 |  |  | 2 |  |
| **Question structure** | | | | | | |
| Question not relevant to specific respondent | 2 | 2 | 2 | 1 | 1 | 2 |
| Respondent skipped relevant question  (paper version only) |  |  |  |  |  | 10* |
| Unclear instruction | 5 | 2 | 6 | 1 | 1 | 2 |
| Unclear branching instructions  (paper version only) |  |  |  |  |  | 7* |
| Question too long | 1 |  |  |  |  |  |
| Complex or awkward syntax | 2 |  | 1 |  | 1 |  |
| Erroneous assumption | 1 | 1 | 1 |  |  |  |
| **RECALL** | | | | | | |
| Undefined reference period | 2 | 4 | 2 | 3 | 1 |  |
| Shortage of memory cues | 1 |  |  |  |  |  |
| High detail required |  | 3 | 5 | 5 |  | 1 |
| **ESTIMATION** | | | | | | |
| Complex estimation | 2 | 1 | 2 |  | 2 |  |
| Potentially sensitive/biasing | 1 |  | 1 |  |  | 1 |
| **RESPONSE SELECTION** | | | | | | |
| Undefined/vague term in response category | 5 | 2 | 2 |  | 2 | 3 |
| Overlapping response categories | 1 | 2 | 2 | 2 |  | 1 |
| Missing response category/categories | 7 | 5 | 4 | 3 | 1 | 1 |
| Excessive response category/categories | 1 | 1 |  | 2 |  | 1 |
| Missing field for comments | 1 | 2 |  |  |  |  |
| **TOTAL** | **37** | **30** | **34** | **18** | **16** | **12** |
| Mean no. of problematic items per patient | 9.25 | 6 | 6.8 | 3 | 4 | 3 |

**Legend:**
Frequency of perceived problems with questionnaire items in the cognitive interview sessions, divided into Tourangeau’s four stages: comprehension, recall, estimation and response selection.

Note that the number of patients participating in the interview sessions ranged from 4-6, and that respondents were presented with a different number of questions due to branching.
*: problems arising in the paper version only. Numbers not included in the total no. of problematic items.
