# Supplementary material for: Driving following defibrillator implantation: development and pilot results from a nationwide questionnaire
Source: BMC Cardiovasc Disord. 2018 Nov 20;18:212. doi: 10.1186/s12872-018-0949-3 (PMC6245910; doi:10.1186/s12872-018-0949-3)
Supplement: Supplementary file 3 — “Danish ICD Patients’ Perspective on Driving – a Nationwide Survey. 2017”. English version (translated from Danish) of the questionnaire specifically constructed for this research project. (PDF 927 kb) [file 12872_2018_949_MOESM3_ESM.pdf]

Danish ICD patients' perspective on driving

# - a nationwide survey

2017

DET SUNDHEDSVIDENSKABELIGE FAKULTET
KØBENHAVNS UNIVERSITET

REGION H Gentofte Hospital

Completing the questionnaire

This questionnaire is divided into three parts:

Part A (blue): To be answered by **all participants**

Part B (green): To be answered **if you have ever experienced an ICD shock**

Part C (purple): To be answered **if you held a professional driver's license** before your ICD implantation

Start by completing part A and continue to part B and/or part C if relevant for you. Please review all your responses before submitting the questionnaire.

General instructions:

1. Please read the question and all response categories before choosing your response
2. Please notice, if you're allowed to tick one or more boxes
3. Please tick the box that describes you best
4. Be aware, that we will ask you questions regarding both the time before and after your ICD implantation
5. Not all questions are relevant for everyone. Keep an eye on the red arrow to the right that guides you through the questionnaire. For example:

**4. Did you hold a valid driver's license for private use (car, motorcycle and tractor) before your ICD implantation?** *(Only one X)*		
Yes	☐	
No	☒	**→ Continue to question 25**

Thank you for your participation!

Part A
To be answered by all participants

Background information

There are many different reasons why a person needs an ICD device. Initially, we would therefore like to ask you some background questions.

In the event that you have had your ICD changed, all questions regarding ICD implantation relates to your first ICD implantation.

1. In general, would you say your health is:

	(Only one X)
Excellent	☐
Very good	☐
Good	☐
Fair	☐
Poor	☐

2. What is the highest level of education that you have completed?

	(Only one X)
Secondary school	☐
Vocational training	☐
High school diploma	☐
Higher education: Short-cycle qualification (up to 3 years)	☐
Higher education: Intermediate-cycle qualification (3 to 5 years)	☐
Higher education: Long-cycle qualification (5 years or more)	☐
Don’t know	☐

3. What was your employment status <u>when you had your ICD implanted</u>?

Please <u>disregard</u> any short sickness leave (< 1 month) leading to your ICD implantation.

	(Only one X)
I was working as an employee / self-employed	☐
I was retired, on early retirement or disability pension (permanently excluded from the work force)	☐
I was unemployed or on long-term sickness leave (temporarily excluded from the work force)	☐
I was studying or in training	☐
If other, please describe what:	

4. Please think back on your life <u>six months before your ICD implantation</u>:

Did you hold a valid driver's license for private use (car, motorcycle and tractor) <u>before your ICD implantation</u>?

	(Only one X)	
Yes	☐	
No	☐	→ **Continue to question 25**

5. Please think back on your life <u>six months before your ICD implantation</u>:

Did you hold a valid driver's license for either professional driving or driving of large vehicles <u>before your ICD implantation</u>?

Large vehicles are defined as small/large trucks and small/large buses (all above 3.5 tonnes)
Professional driving is defined as driving for professional purposes, including any passenger transportation.

	(Only one X)	
Yes	☐	→ **Remember to complete part C**
No	☐	

6. Did anyone else in your household hold a valid driver's license at the time of your ICD implantation?

	(Only one X)
Yes	☐
No	☐

7. Have you ever experienced one or more shocks from your ICD?

	(Only one X)	
Yes	☐	→ Remember to complete part B
No	☐	

Driving habits

In the following questions, we would like to know more about your driving habits. We will ask questions into your driving habits both before and after you had your ICD implanted.

We are only interested in driving situations where you were the driver of the vehicle (car, motorcycle, tractor, bus or truck).

8. If you think back on you driving habits during the six months leading up to your ICD implantation: On which occasions did you typically drive?

	(You may select more than one answer)	
Professional driving or driving of large vehicles during work hours	☐	
During work hours (fx. as a salesman, farmer, craftsman)	☐	
To/from work or school/university	☐	
In relation with practical errands (fx. shopping, visits to the doctor)	☐	
To visit family and friends	☐	
In relation with leisure activities (fx. exercise, theater, holiday)	☐	
I did not drive during the period	☐	→ Continue to question 10

9. If you think back on you driving habits during the six months leading up to your ICD implantation: How many hours a week did you typically drive?

(Only one X)

I drove less than 1 hour a week	☐
I drove 1-3 hours a week	☐
I drove 4-6 hours a week	☐
I drove 7-9 hours a week	☐
I drove 10 hours a week or more	☐

10. Have you resumed private driving after your ICD implantation?

(Only one X)

Yes	☐	
No	☐	→ **Continue to question 18**
If no, please elaborate on why you haven't resumed driving:		

10.1. How long after your ICD implantation did you drive for the first time?

(Write a number before either days/weeks/months)

☐☐	Days
☐☐	Weeks
☐☐	Months

Current driving habits:

11. If you think about your driving habits during the last month:
How many hours a week do you typically drive?

(Only one X)

I haven't driven during the last month	☐
I drive less than 1 hour a week	☐
I drive 1-3 hours a week	☐
I drive 4-6 hours a week	☐
I drive 7-9 hours a week	☐
I drive 10 hours a week or more	☐

Cardiac symptoms while driving

In the following questions we will ask you, if you've experienced any cardiac symptoms while driving, after you had your ICD implanted.

We are only interested in driving situations where you were the driver of the vehicle (car, motorcycle, tractor, bus or truck).

Remember that your responses are treated completely anonymously and will not be disclosed to other health care professionals or the authorities.

12. Have you, after you had your ICD, experienced one or more of the following symptoms while driving? If yes, how many times?

	(You may select more than one)	*(If yes, how many times?)*	
Received an ICD shock while driving	☐	☐☐	→ **Continue to question 13**
Lost consciousness while driving (not related to an ICD shock)	☐	☐☐	→ **Continue to question 14**
Experienced dizziness while driving	☐	☐☐	→ **Continue to question 15**
Experienced palpitations while driving	☐	☐☐	→ **Continue to question 16**
Experienced chest pain while driving	☐	☐☐	→ **Continue to question 17**
None of the above	☐		→ **Continue to question 18**

In the following two questions, we would like to know more about your experiences receiving an ICD shock while driving.

13. Have you lost consciousness in conjunction with receiving an ICD shock while driving? If yes, how many times?

	(Only one X)	*(If yes, how many times?)*
Yes	☐	☐☐
No	☐	

13.1. Have you been involved in a motor vehicle accident in conjunction with an ICD shock while driving? If yes, how many times?

A motor vehicle accident is defined by an accident leading to either material damage and/or physical injury.

	(Only one X)	*(If yes, how many times?)*
Yes	☐	☐☐
No	☐	

14. Have you been involved in a motor vehicle accident in conjunction with loss of consciousness while driving? If yes, how many times?

A motor vehicle accident is defined by an accident leading to either material damage and/or physical injury.

	(Only one X)	*(If yes, how many times?)*
Yes	☐	☐☐
No	☐	

In the following two questions, we would like to know more about your experiences with dizziness while driving.

15. Have you, after your ICD implantation, had to stop the vehicle due to dizziness?

	(Only one X)
Yes	☐
No	☐

15.1. Have you been involved in a motor vehicle accident in conjunction with dizziness while driving? If yes, how many times?

A motor vehicle accident is defined by an accident leading to either material damage and/or physical injury.

	(Only one X)	*(If yes, how many times?)*
Yes	☐	☐☐
No	☐	

In the following two questions, we would like to know more about your experiences with palpitations while driving.

16. Have you, after your ICD implantation, had to stop the vehicle due to palpitations?

	(Only one X)
Yes	☐
No	☐

16.1. Have you been involved in a motor vehicle accident in conjunction with palpitations while driving? If yes, how many times?

A motor vehicle accident is defined by an accident leading to either material damage and/or physical injury.

	(Only one X)	*(If yes, how many times?)*
Yes	☐	☐☐
No	☐	

In the following two questions, we would like to know more about your experiences with chest pain while driving.

17. Have you, after your ICD implantation, had to stop the vehicle due to chest pain?

	(Only one X)
Yes	☐
No	☐

17.1. Have you been involved in a motor vehicle accident in conjunction with chest pain while driving? If yes, how many times?

A motor vehicle accident is defined by an accident leading to either material damage and/or physical injury.

	(Only one X)	*(If yes, how many times?)*
Yes	☐	☐☐
No	☐	

Information on driving following ICD implantation

It may vary from person to person, whether you're allowed to drive following ICD implantation. Health professionals are responsible for giving you thorough and adequate information on driving following ICD implantation.

In order to improve the quality of the information, we will ask you some questions on your experience with information on driving following ICD implantation. In the event that you have had your ICD changed, all questions relate to your first ICD implantation.

18. Please think back to the hospitalization during which you had your ICD implanted.

Did you receive any information from health care professionals on private driving following ICD implantation?

	(Only one X)	
Yes	☐	
No	☐	→ **Continue to question 20**
Cannot remember	☐	→ **Continue to question 20**

18.1 How did you receive the information?

	(Only one X)
Both verbally and in writing	☐
Only verbally	☐
Only in writing	☐

18.2. What information did you receive about private driving following ICD implantation?

	(Only one X)
That I could resume driving immediately	☐
That I could resume driving when I felt ready	☐
That I could resume driving after 1 week	☐
That I could resume driving after 1 month	☐
That I could resume driving after 3 months	☐
That I could resume driving after 6 months	☐
That I could never drive again	☐
If other, please describe what:	

19. How satisfied are you with the quality of the information given on driving following ICD implantation?

(Only one X)

Very satisfied	Satisfied	Neutral	Dissatisfied	Very dissatisfied	Not relevant
☐	☐	☐	☐	☐	☐

Please note any comments or suggestions for improvement on information about driving following ICD implantation below:

Living with an ICD

The following questions relates to how potential driving restrictions following ICD implantation and/or ICD shock may influence one's daily life activities and well-being.

20. How do you agree with the following statements:
As a result of having an ICD …

(One X in each line)	Strongly agree	Agree	Uncertain	Disagree	Strongly disagree
… I'm nervous about driving	☐	☐	☐	☐	☐
… I'm afraid that I'll have an ICD shock while driving	☐	☐	☐	☐	☐
… I try to avoid highways	☐	☐	☐	☐	☐
… I try to avoid driving alone	☐	☐	☐	☐	☐
… I try to avoid driving with children	☐	☐	☐	☐	☐

If you have altered your driving habits in any other ways as a result of having an ICD, you may write it below:

The following four questions (no. 21-24) are only for persons that have been subject to a minimum of 1 month driving restrictions following either ICD implantation or ICD shock.

Everyone else should proceed to question 25

21. Please think back to <u>periods with driving restrictions</u> following ICD implantation and/or ICD shock:

Have driving restrictions <u>following ICD implantation and/or ICD shock</u> impeded with your daily life?

(Only one X)

Not at all	A little	Moderately	Very	Extremely
☐	☐	☐	☐	☐

22. Have you, as a result of driving restrictions <u>following ICD implantation and/or ICD shock</u>, felt restricted in the activities below? If so, how much?

(One X in each line)	Yes, very limited	Yes, somewhat limited	No, not limited at all	Not relevant
Maintaining paid employment	☐	☐	☐	☐
Getting to/from work/school	☐	☐	☐	☐
Running errands (fx. shopping, visits to the doctor)	☐	☐	☐	☐
Meeting family and friends	☐	☐	☐	☐
Engaging in leisure activities (fx. exercise, theater, holiday)	☐	☐	☐	☐

23. How do you agree with the following five statements:

In periods with driving restrictions…

(One X in each line)	Strongly agree	Agree	Uncertain	Disagree	Strongly disagree
… I have felt like a burden on my surroundings	☐	☐	☐	☐	☐
… I have found the driving restrictions annoying	☐	☐	☐	☐	☐
… the driving restrictions have affected me in a positive way	☐	☐	☐	☐	☐
… the driving restrictions have given me a bad mood	☐	☐	☐	☐	☐
… it has been difficult to adhere with the driving restrictions	☐	☐	☐	☐	☐

24. During periods with driving restrictions, have you driven anyway?

Remember that your responses are treated completely anonymously and will not be disclosed to other health care professionals or the authorities.

	(Only one X)
Yes	☐
No	☐
Not relevant / no comment	☐

If you have any comments, please write them below:

25. Is there anything else you would like us to know?

If you have ever experienced an ICD shock → **Continue to part B**

If you held a professional/large vehicle driver's license before your ICD implantation → **Continue to part C**

If you neither have experienced an ICD shock nor held a professional/large vehicle driver's license before your ICD implantation, you have completed the questionnaire!

Please review all your responses before submitting the questionnaire in the enclosed envelope. The postage is pre-paid.

Thank you again for your participation!

Part B
To be answered if you have ever experienced an ICD shock

Think back on the time/times you experienced an ICD shock/shocks.

1. Was/were the ICD shock/shocks:

	(Only one X)	
Appropriate,<br>ie. to terminate a life-threatening arrhythmia	☐	
Inappropriate<br>fx. a misinterpretation of your heart rhythm	☐	→ Continue to question 5
I've experienced both appropriate and inappropriate ICD shocks	☐	
Don't know	☐	

2. When did you experience your most recent ICD shock?

	(Only one X)
Less than 3 months ago	☐
More than 3 months ago	☐

3. Think back to the first time you experienced an appropriate ICD shock, that is, an ICD shock given to terminate a life-threatening arrhythmia.

Have you resumed driving following your first appropriate ICD shock?

	(Only one X)	
Yes	☐	
No	☐	→ Continue to question 4

3.1. How long after the appropriate ICD shock did you resume driving?

(Write a number before either days/weeks/months)

☐☐	Days
☐☐	Weeks
☐☐	Months

4. Think back to the first time you experienced an appropriate ICD shock, that is, an ICD shock given to terminate a life-threatening arrhythmia.

Did you receive any information from health care professionals on driving following ICD shock?

	(Only one X)	
Yes	☐	
No	☐	**→ Continue to question 5**
Cannot remember	☐	**→ Continue to question 5**

4.1. How did you receive the information?

	(Only one X)
Both verbally and in writing	☐
Only verbally	☐
Only in writing	☐

4.2 What information did you receive about driving following ICD shock?

	(Only one X)
That I could resume driving immediately	☐
That I could resume driving when I felt ready	☐
That I could resume driving after 1 week	☐
That I could resume driving after 1 month	☐
That I could resume driving after 3 months	☐
That I could resume driving after 6 months	☐
That I could never drive again	☐

If other, please describe what:

5. How satisfied are you with the quality of the information given on driving following ICD shock?

(Only one X)

Very satisfied	Satisfied	Neutral	Dissatisfied	Very dissatisfied	Not relevant
☐	☐	☐	☐	☐	☐

Please note any comments or suggestions for improvement on information about driving following ICD shock below:

If you held a professional/large vehicle driver's license before your ICD implantation **→ Continue to part C**

If you didn't hold a professional/large vehicle driver's license before your ICD implantation, you have completed the questionnaire!

Please review all your responses before submitting the questionnaire in the enclosed envelope. The postage is pre-paid.

Thank you again for your participation!

Part C
To be answered if you held a professional/large vehicle driver's license before your ICD implantation

1. For which profession did you use your professional/large vehicle driver's license <u>before your ICD implantation</u>?

(Only one X)

Truck driver	☐
Bus driver	☐
Taxi driver	☐
If other, please describe what:	

2. Did you utilize your professional/large vehicle driver's license <u>during the six months before your ICD implantation</u>?

(Only one X)

Yes	☐
No	☐

3. Have you resumed professional driving or driving of large vehicles <u>following your ICD implantation</u>?

(Only one X)

Yes	☐	
No	☐	→**Continue to question 4**

3.1. How long <u>after your ICD implantation</u> did you resume professional driving or driving of large vehicles?

(Write a number before either days/weeks/months)

☐☐	Days
☐☐	Weeks
☐☐	Months

4. Please think back to the <u>hospitalization</u> during which you had your ICD implanted.

Did you receive any information from health care professionals on professional driving or driving of large vehicles <u>following ICD implantation</u>?

	(Only one X)	
Yes	☐	
No	☐	→ **Continue to question 5**
Cannot remember	☐	→ **Continue to question 5**

4.1. How did you receive the information?

	(Only one X)
Both verbally and in writing	☐
Only verbally	☐
Only in writing	☐

4.2. What information did you receive about professional driving or driving of large vehicles <u>following ICD implantation</u>?

	(Only one X)
That I could resume professional/large vehicle driving immediately	☐
That I could resume professional/large vehicle driving when I felt ready	☐
That I could resume professional/large vehicle driving after <u>1 week</u>	☐
That I could resume professional/large vehicle driving after <u>1 month</u>	☐
That I could resume professional/large vehicle driving after <u>3 months</u>	☐
That I could resume professional/large vehicle driving after <u>6 months</u>	☐
That I <u>never</u> could drive professionally or large vehicles again	☐
If other, please describe what:	

5. Did you lose your job due to professional/large vehicle driving restrictions following ICD implantation?

	(Only one X)
Yes	☐
No	☐
Not relevant / no comment	☐

If you have any comments, you can write them below:

6. How satisfied are you with the quality of the information given on <u>professional driving or driving of large vehicles</u> following ICD implantation?

(Only one X)

Very satisfied	Satisfied	Neutral	Dissatisfied	Very dissatisfied	Not relevant
☐	☐	☐	☐	☐	☐

Please note any comments or suggestions for improvement on information on professional driving or driving of large vehicles after ICD implantation below:

You have completed the questionnaire!

Please review all your responses before submitting the questionnaire in the enclosed envelope. The postage is pre-paid.

Thank you again for your participation!
