# Supplementary material for: Driving following defibrillator implantation: development and pilot results from a nationwide questionnaire
Source: BMC Cardiovasc Disord. 2018 Nov 20;18:212. doi: 10.1186/s12872-018-0949-3 (PMC6245910; doi:10.1186/s12872-018-0949-3)
Supplement: Supplementary file 4 — Table S3. Test-Retest Agreement. (DOCX 19 kb) [file 12872_2018_949_MOESM4_ESM.docx]

Additional file 4: **Table S3** Test-Retest Agreement

| **Questionnaire item** | **Number of pilot test respon-dents** | **Number of retest respon-dents** | **Kappa, weighted kappa (*) or Intraclass Correlation Coefficient (^§^)** |
| --- | --- | --- | --- |
| **Background information** | | | |
| Self-assessed health | 39 | 19 | 0.63* |
| Educational attainment | 39 | 19 | 0.83* |
| Employment status at ICD implantation | 37 | 19 | 0.71 |
| Private (Group 1) driver’s license | 38 | 19 | 1.00 |
| Professional (Group 2) driver’s license | 37 | 19 | 1.00 |
| Type of professional driver’s license: Truck driver | 10 | 7 | 0.64 |
| Type of professional driver’s license: Bus driver | 10 | 7 | 1.0 |
| Type of professional driver’s license: Taxi driver | 10 | 7 | 0 |
| Type of professional driver’s license: Other | 10 | 7 | 1.0 |
| Use of professional driver’s license within six months before ICD implantation | 10 | 7 | 0.80 |
| Other drivers in the household | 37 | 19 | 0.23 |
| Previous ICD shock | 37 | 19 | 1.00 |
| Type of previous ICD shock | 5 | 2 | 0.73 |
| Last ICD shock more or less than three months ago | 5 | 2 | 1.00 |
| Occasions for driving prior to ICD implantation | | | |
| Professional driving | 37 | 19 | 0.64 |
| During work hours | 37 | 19 | 0.50 |
| To /from work/school | 37 | 19 | 0.57 |
| Practical errands | 37 | 19 | 0.30 |
| Social occasions | 37 | 19 | 0.21 |
| Hobbies | 37 | 19 | 0.55 |
| Did not drive during the period | 37 | 19 | 0.64 |
| Hours driving/week prior to ICD implantation | 35 | 18 | 0.65* |
| Resumption of private driving after ICD implantation | 37 | 19 | 1.00 |
| Time to resumption of private driving after ICD implantation | 35 | 17 | 0.98^§^ |
| Resumption of professional driving following ICD implantation | 10 | 7 | 1.00 |
| Time to resumption of professional driving after ICD implantation | 1 | 1 | NA^§^ |
| Resumption of driving following ICD shock (appropriate) | 4 | 2 | 1.00 |
| Time to resumption of driving after ICD shock (appropriate) | 4 | 2 | 1.00^§^ |
| Current hours driving/week | 35 | 17 | 0.63 |
| **Cardiac symptoms while driving** | | | |
| Received an ICD shock while driving | 35 | 17 | 1.00 |
| Lost consciousness while driving | 35 | 17 | 1.00 |
| Dizziness while driving | 35 | 17 | 1.00 |
| Palpitations while driving | 35 | 17 | 0.64 |
| Palpitations that necessitated stopping the vehicle | 4 | 1 | 0.64 |
| Palpitations that resulted in a motor vehicle accident | 4 | 1 | 0.64 |
| Chest pain while driving | 35 | 17 | 1.00 |
| No cardiac symptoms while driving | 35 | 17 | 0.83 |
| **Information on driving restrictions** | | | |
| Received information on private driving | 37 | 17 | 0.76 |
| Mode of delivering information on private driving | 18 | 6 | 0.69 |
| What information received on private driving | 18 | 6 | 0.70 |
| Satisfaction with information on private driving | 18 | 6 | 0.54* |
| Received information on professional driving | 9 | 7 | 0.80 |
| Mode of delivering information on professional driving | 1 | 1 | 1.0 |
| What information received on professional driving | 1 | 1 | 1.0 |
| Satisfaction with information on professional driving | 1 | 1 | 1.0* |
| Received information on driving following appropriate ICD shock | 4 | 2 | 1.0 |
| Mode of delivering information on driving following appropriate ICD shock | 3 | 1 | 1.0 |
| What information received on driving following appropriate ICD shock | 3 | 1 | 1.0 |
| Satisfaction with information on driving following appropriate ICD shock | 3 | 1 | 1.0* |
| **Living with an ICD** | | | |
| Nervous about driving | 34 | 17 | 0.75* |
| Afraid of having an ICD shock while driving | 34 | 17 | 0.79* |
| Try to avoid highways | 34 | 17 | 0.77* |
| Try to avoid driving alone | 34 | 17 | 0.73* |
| Try to avoid driving with children | 34 | 17 | 0.73* |
| Driving restrictions’ level of impediment with daily life | 11 | 5 | 0.51* |
| Restrictions in maintaining employment | 9 | 3 | 0.34* |
| Restrictions in getting to/from work | 9 | 3 | 0.31* |
| Restrictions in running errands | 12 | 5 | 0.29* |
| Restrictions in social life | 12 | 5 | 0.40* |
| Restrictions in leisure activities | 12 | 5 | 0.34* |
| Have felt like a burden on surroundings due to driving restrictions | 12 | 5 | 0.49* |
| Have found the driving restrictions annoying | 12 | 5 | 0.34* |
| Have been positively affected by driving restrictions | 12 | 5 | 0.31* |
| Have been in a bad mood due to the driving restrictions | 12 | 5 | 0.47* |
| Have found it difficult to adhere to the driving restrictions | 12 | 5 | 0.24* |
| Have knowingly been driving although restricted | 12 | 5 | 0.50 |
| Loss of job due to professional driving restrictions | 9 | 7 | 0.70 |

**Legend:**
Kappas, weighted kappas (*) and intraclass correlation coefficients (^§^) of test-retest variables. Commonly accepted interpretations of kappa statistics state that values 0.81-1 represent almost perfect agreement; 0.61-0.80 substantial agreement; 0.41-0.60 moderate agreement; 0.21-0.40 fair agreement; 0.01-0.20 slight agreement; and <0 less than chance agreement. For intraclass correlation coefficients, large values (close to 1) indicate low random error variability and thus high agreement.
